# Autophagy Limits Endotoxemic Acute Kidney Injury and Alters Renal Tubular Epithelial Cell Cytokine Expression

**DOI:** 10.1371/journal.pone.0150001

**Published:** 2016-03-18

**Authors:** Jeremy S. Leventhal, Jie Ni, Morgan Osmond, Kyung Lee, G. Luca Gusella, Fadi Salem, Michael J. Ross

**Affiliations:** 1 Division of Nephrology, Department of Medicine, Icahn School of Medicine at Mount Sinai, New York, New York, United States of America; 2 Renal Division, James J Peters Bronx VA Medical Center, Bronx, New York, United States of America; 3 Department of Pathology, Icahn School of Medicine at Mount Sinai, New York, New York, United States of America; National Cancer Institute, UNITED STATES

## Abstract

Sepsis related acute kidney injury (AKI) is a common in-hospital complication with a dismal prognosis. Our incomplete understanding of disease pathogenesis has prevented the identification of hypothesis-driven preventive or therapeutic interventions. Increasing evidence in ischemia-reperfusion and nephrotoxic mouse models of AKI support the theory that autophagy protects renal tubular epithelial cells (RTEC) from injury. However, the role of RTEC autophagy in septic AKI remains unclear. We observed that lipopolysaccharide (LPS), a mediator of gram-negative bacterial sepsis, induces RTEC autophagy *in vivo* and *in vitro* through TLR4-initiated signaling. We modeled septic AKI through intraperitoneal LPS injection in mice in which autophagy-related protein 7 was specifically knocked out in the renal proximal tubules (ATG7KO). Compared to control littermates, ATG7KO mice developed more severe renal dysfunction (24hr BUN 100.1mg/dl +/- 14.8 vs 54.6mg/dl +/- 11.3) and parenchymal injury. After injection with LPS, analysis of kidney lysates identified higher IL-6 expression and increased STAT3 activation in kidney lysates from ATG7KO mice compared to controls. *In vitro* experiments confirmed an altered response to LPS in RTEC with genetic or pharmacological impairment of autophagy. In conclusion, RTEC autophagy protects against endotoxin induced injury and regulates downstream effects of RTEC TLR4 signaling.

## Introduction

Septic AKI is an important clinical problem and is characterized by acute RTEC injury occurring in the setting of severe infection, which is often accompanied by increased systemic levels of bacteria-derived molecules, including LPS [1]. The kidneys receive approximately 20% of cardiac output despite comprising only 1% of body weight [2] and are therefore exquisitely sensitive to blood borne molecules.

RTEC in the proximal tubule are directly exposed to circulating LPS via glomerular filtrate, and filtered LPS is bound and internalized via TLR4 on the luminal RTEC membrane [3]. LPS can induce both pro-death and pro-survival cellular pathways, the balance of which determines whether cells are irreversibly injured or are protected from death and eventually regain normal function [4]. Strategies to enhance activation of cytoprotective cellular processes may therefore allow novel therapeutic approaches to prevent and/or treat septic AKI.

Autophagy is an adaptive intracellular response to starvation and cellular stress with notable relationships to health and disease [5]. Kidney-related research has focused on autophagy in RTEC and podocytes. In the podocyte, absence of autophagy causes glomerulosclerosis and proteinuria in aged mice, but has no acute defects upon glomerular development or function [6]. Similarly, pan-tubular knockout of autophagy related protein 5 (ATG5), an important mediator of autophagic vesicle formation, produces only modest changes in renal function without pathological evidence in the corresponding histology.[7] Tubular segment specific deletion of ATG5, either in distal or proximal tubule, has no measurable effect on glomerular filtration rate [8] [7]. In contrast to its modest effects on renal homeostasis in unstressed mice, the inhibition of autophagy has been demonstrated to have dramatic effects in the setting of acute kidney injury (AKI). While early *in vitro* models yielded conflicting data, pharmacological inhibition of autophagy with either 3-methyladenine (3MA) or chloroquine worsened ischemia/reperfusion injury (IRI) [9]. Subsequently, conditional knockout models confirmed that tubular deletion of autophagy specific genes (i.e. *Atg5* or *Atg7*) predisposed mice to worsened IRI and cisplatin-mediated kidney injury [7, 8, 10, 11].

In contrast to the genetic evidence in cisplatin and IRI models, the role of autophagy in models of septic AKI remains unclear. Published data suggest that autophagy is induced primarily in the proximal tubule during the cecal ligation and puncture (CLP) model of septic AKI [12]. Indirect activation of autophagy by administration of a mammalian target of rapamycin (mTOR) inhibitor or the adenosine monophosphate associated kinase (AMPK) activator 5-aminoimidazole-4-carboxamide ribonucleotide (AICAR) ameliorates AKI in the LPS and CLP models, respectively [13]. Accordingly, in vivo siRNA-mediated knock down of phosphatidylinositol 3-kinase catalytic subunit type 3 (Vps34), which is involved in multiple intracellular transport functions and is necessary for autophagy induction, worsened LPS-induced AKI [13]. However, whether worsened AKI results from suppression of tubular autophagy or dysregulation of other intracellular pathways unrelated to autophagy remains unclear and requires modeling with a deletion of an autophagy specific gene.

In the following studies, we demonstrate 1) that LPS induces autophagy in RTEC 2) that this effect is mediated via Toll-like receptor 4 (TLR4), and 3) that induction of autophagy is necessary to limit the severity of AKI in the LPS-induced model of AKI. Furthermore, we demonstrate enhanced innate immune response in murine RTEC with absent or impaired autophagy. Through the use of a transgenic mouse model with tubular specific deletion of autophagy protein 7 (ATG7), we definitively establish the renoprotective effect of tubular autophagy in LPS-induced AKI thereby identifying autophagy as a novel target for preventing or treating septic AKI.

## Methods

### Ethics Statement

All animal studies were performed under a protocol (#2013–1397) approved by the Mount Sinai Institutional Animal Care and Utilization Committee (IACUC) in adherence to NIH guidelines of animal care. In accordance with our protocol, mice were monitored daily for signs of distress (markedly decreased or absent food/water intake, signs of dehydration, unresponsive to extraneous activity/stimuli, persistently "hunched", exhibiting labored respirations, persistent tremors, convulsions, or self-mutilation) and would have been euthanized early should these signs have appeared. None of the mice died prior to the experimental endpoint. Mice were euthanized via controlled gradual (~10%/minute) displacement with carbon dioxide using a flow meter, consistent with our institutional and the most recent American Veterinary Medicine Association (AMVA) guidelines on euthanasia.

### Mouse Studies

Ten to twelve-week-old C57BL/6, C57Bl/10ScN, *PEPCK-Cre; ATG7*^*flox/flox*^, and *ATG7*^*flox/flox*^ mice were injected *i*.*p*. with 15mg/kg Ultrapure LPS (*E*. *coli* 0111:B4, Invivogen) or sterile saline. In experiments where blood urea was to be measured, serum (less than 20 microliters) was drawn prior to injection, 24 hours post-injection, and then at time of sacrifice. Kidneys were harvested at 48hr and protein/RNA was isolated from the cortex. Kidneys from LPS and saline-injected GFP-LC3 mice, which express a GFP-LC3 fusion protein under control of the constitutive CAG promoter [14], were first fixed in 2% paraformaldehyde, incubated in a sucrose gradient and blocked in OCT media for frozen sectioning. Images were acquired on a Zeiss AxioImager using 63X and 100X oil immersion lenses with z-stacking and maximal intensity projections. Urea measurements were performed using the Quantichrom Urea Assay Kit (BioAssay Systems) per manufacturer’s instructions. Blinded pathology scoring of slides from LPS injected animals was performed using a scale from 0 to 3 for a composite of indicators including tubular dilatation, vacuolization, degeneration, brush border loss, and cell death as previously described [15]. A score of 0 was assigned if <5% of tubules were affected, 1 if 5–33% of tubules were affected, 2 if 34–66% of tubules were affected and 3 if 67%-100% of tubules were affected. Scores between groups were compared using a Fisher Exact Test and significance defined as p<0.05.

### Cell Culture

RTEC cell lines (HPT1b and FKT) and primary cultures from C57Bl/10ScN and C57Bl/6 kidneys were grown in a 50:50 mixture of DMEM/HAM’S F-12 (ATCC) supplemented with 2% FBS (Invitrogen), 5pM tri-iodothyronine (Sigma), insulin-transferrin-selenium supplement(Sigma), 3.5micrograms/mL ascorbic acid, 25ng/mL prostaglandin E1, 25ng/mL hydrocortisone, and 10ng/mL epidermal growth factor. Floxed Kidney Tubular (FKT) cell line was created after isolating RTEC from *ATG7*^*flox/flox*^ mice. Briefly, cortex was isolated and minced with sterile blades and then filtered through a 40-micron filter before being pelleted and then resuspended in defined media. RTEC on day 5 were recognizable by their characteristic polygonal/cobblestone morphology, refractive nature, and the tendency to form domes in areas of confluence. Cells were immortalized via transduction with the lentivector VVPW/mTert that constitutively expresses the mouse telomerase [16]. A subset of FKT cells were transduced with either VPB/Cre, expressing the Cre recombinase, or the empty blasticidin-selectable VPB lentivector [16]. Following blasticidin selection, we established the FKT+ cells expressing the wild-type Atg7 and the FKT- variant cell line in which the Atg7 gene was ablated.

### Western blotting

Western blotting was conducted as previously described [17] using anti-LC3 (MBL), anti-ATG7(Cell Signal), anti-STAT3 (Cell Signal), anti-phospho-STAT3 (Cell Signal), anti beta actin (Sigma), and anti-GAPDH as primary antibodies and HRP-conjugated donkey anti-rabbit (Jackson Laboratory) as secondary antibody. Quantification of band intensity was performed using Image Studio (Licor).

### Immunofluorescence

Localization of LC3 by immunofluorescence microscopy was performed as described [18] using anti-LC3 (MBL) as primary antibody and Cy3 goat anti-rabbit (Jackson ImmunoResearch) as secondary antibody.

### Quantitative real-time PCR

Kidneys from injected mice were snap frozen in liquid nitrogen at the time of sacrifice. Subsequently, small pieces of frozen cortex were immersed and homogenized in TRI REAGENT (Molecular Research Center) with a benchtop homogenizer. RNA was isolated using Direct-zol RNA kit (Zymo) and quantified using a Nanodrop spectrophotometer (Thermo Scientific). One microgram of total RNA was reverse transcribed using Maxima First Strand cDNA Synthesis Kit for RT-qPCR (ThermoFisher Scientific. cDNA was analyzed by quantitative real time PCR (qPCR) using Absolute Blue qPCR Mix (ThermoFisher Scientific) and previously published primers for inflammation related cytokines[15] on a CFX 384 Touch Real-Time PCR Detection System (Bio-Rad). Gene expression was first normalized to cyclophilin A (housekeeping control) and then calculated using the 2^(-deltadeltaCT)^ method [19, 20].

### Statistical analyses

Urea concentrations between groups at each time-point were compared using a Student’s t-test and expressed as means +/- SEM. For qPCR analysis, differences in fold-change were first compared using a Kruskal-Wallis test when there were more than two groups (Microsoft excel). Subsequent pair-wise comparisons were performed with a Mann-Whitney Test to detect significant differences between individual groups with a p<0.05 considered significant.

## Results

### LPS Induces Autophagy in Tubular Epithelial Cells

**Fig 1:**
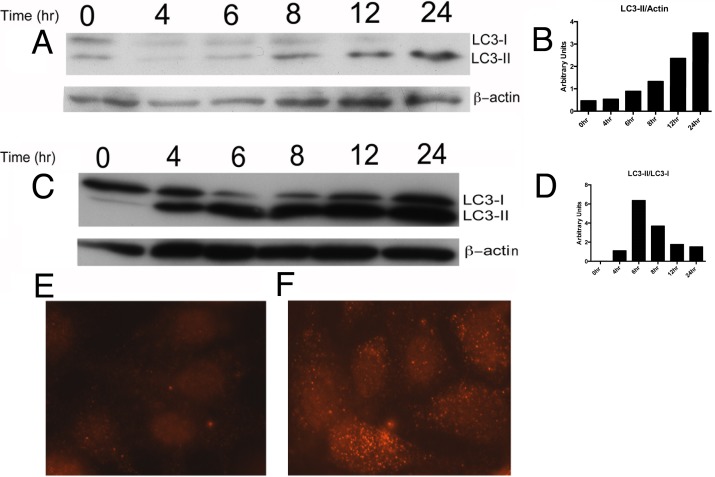
LPS Induces Autophagy in RTEC: Western Blot for LC3 in HPT1b cells incubated over increasing time with 1 microgram /mL LPS in the absence (A and B) or presence of 100nM Baf A1 (C and D). LPS-induced autophagy was confirmed by immunofluorescence staining of LC3 in HPT1b cells before (E) and after (F) 24-hour treatment with 1 microgram /mL LPS.

To determine if LPS induces autophagy in RTEC, we incubated HPT1b cells with 1microgram/ml LPS and subsequently collected cell lysates at several time points. HPT1b is a clonal cell line derived from the previously characterized HPT1 human proximal tubular epithelial cell (PTEC) line [18, 21]. Cell lysates were evaluated for abundance of LC3-II, the lipidated form of LC3-I that indicates autophagosome formation and remains associated with autophagosomes after their maturation. Cells incubated with LPS demonstrated a progressive accumulation of LC3-II (Fig 1A and 1B) relative to beta-actin (loading control). To determine whether the LPS-induced increase of LC3-II abundance in HPT1b cells was due to increased autophagosome formation or to decreased autophagosome degradation, we performed the experiment in the presence of the lysosomal inhibitor bafilomycin A1. In the presence of bafilomycin A1, LPS induced a more rapid increase in LC3-II abundance relative to LC3-I (Fig 1C and 1D) confirming that LPS increased autophagy within four hours in HPT1b cells.

To further examine the autophagic response of RTEC to LPS, we visualized LC3 by immunofluorescence microscopy in HPT1b cells after incubation with 1 microgram /ml LPS. The accumulation of LC3-positive puncta is a commonly used indicator of autophagosome formation. After twenty-four hours, LC3 remained faint and diffusely distributed in control cells (Fig 1E) whereas LPS-treated cells demonstrated a prominent increase in the presence of bright LC3-positive puncta, representing nascent autophagosomes (Fig 1F).

**Fig 2:**
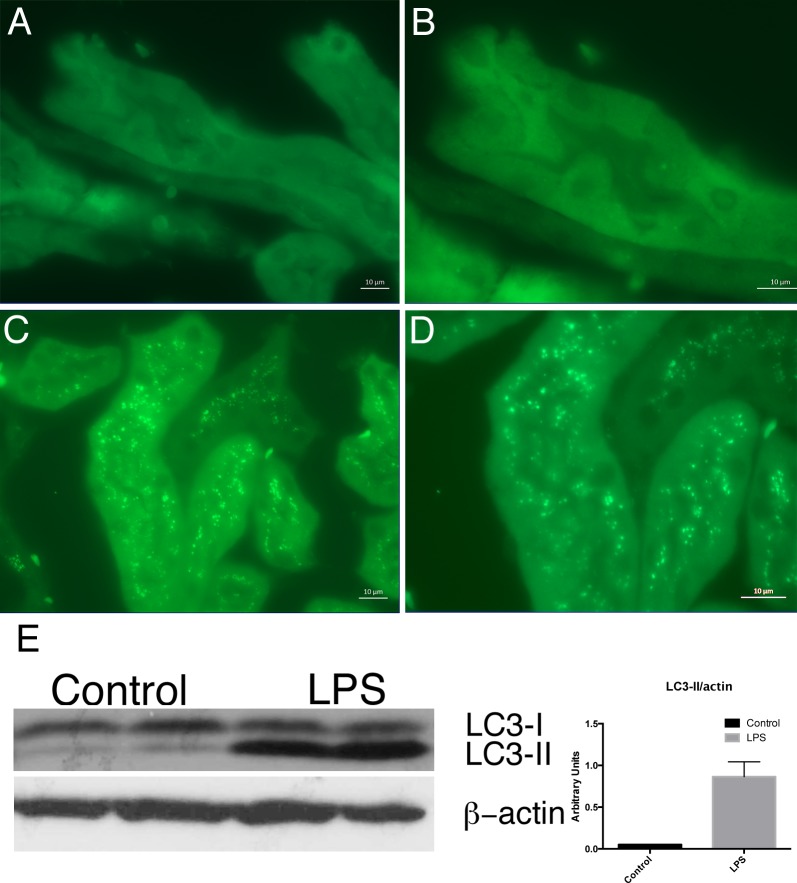
LPS Induces Autophagy in RTEC *in vivo*: GFP-LC3 reporter mice demonstrate diffuse faint GFP fluorescence in RTEC of control-injected mice (A,B; 630X and 1000X, respectively). LPS injection induced marked increase in abundance of green fluorescent puncta in RTEC (C,D; 630X and 1000X, respectively). Western blot for LC3 demonstrates increased LC3-II accumulation in LPS-treated as compared to control injected mice (E).

To determine whether LPS similarly induced autophagy in RTEC *in vivo*, we utilized transgenic autophagy reporter mice that express a GFP-LC3 fusion protein under the control of the beta-actin promoter. These mice allow for the identification of autophagosomes in all cells by assessing for change in localization of green fluorescence from diffuse to punctate[14]. RTEC in control-injected mice displayed a homogenous pattern of fluorescence, consistent with low levels of basal autophagy (Fig 2A and 2B). However, RTEC in LPS-injected mice demonstrated markedly increased formation of fluorescent puncta, indicating that LPS injection induced autophagy in RTEC *in vivo* (Fig 2C and 2D). These results were confirmed by western blotting of kidney protein lysates from LPS-injected mice, which demonstrated increased LC3-II abundance compared to control-injected mice (Fig 2E). Taken together, these data demonstrate that LPS induces autophagy in RTEC *in vivo*.

### LPS-induced Autophagy in RTEC is TLR4 Dependent

**Fig 3:**
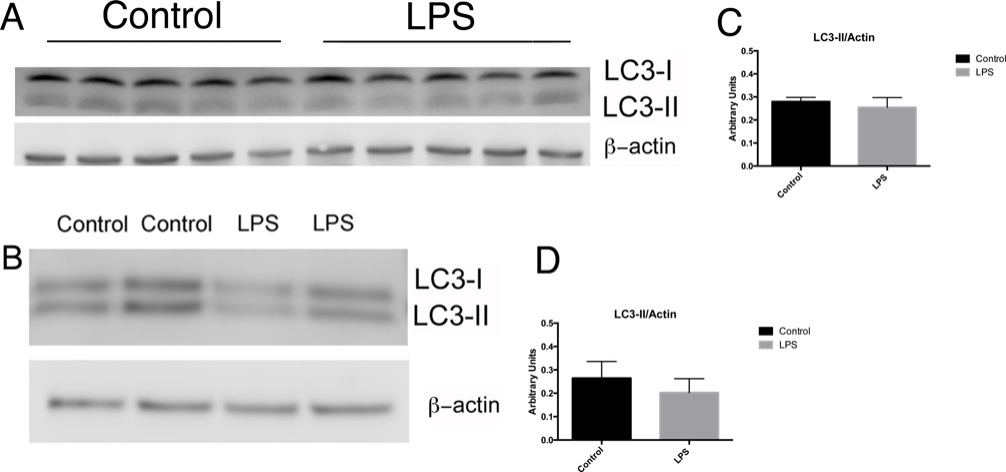
LPS-induced autophagy is TLR4 dependent: Western blot for LC3 and corresponding quantification in lysates from renal cortex of C57BL/10ScN mice 24hr after injection with 15mg/kg LPS or control sterile saline demonstrated no increased autophagy (A, C). Immunodetection of LC3 in primary RTEC isolated from C57BL/10ScN mice grown in the absence or presence of 1 microgram /mL LPS for 24 hours (B, D) revealed no differences in the abundance or distribution of LC3 isoforms.

Canonical signaling through Toll-like Receptor 4 (TLR4) is initiated by its ligand, LPS [22]. TLR4 is expressed on cells of the proximal tubule and mediates tubular responses to LPS [3]. However, proximal tubule cells can internalize LPS, even in the absence of TLR4, via flow-mediated endocytosis[3]. To determine whether TLR4 signaling is required for LPS-induced autophagy, we injected C57Bl/10ScN mice, which possess no functional TLR4, with 15mg/kg LPS. Twenty-four hours later, renal cortices from treated and control mice were harvested and LC3-II expression determined by western blotting. No differences in LC3-II accumulation were detectable in C57Bl\10ScN mice injected with LPS compared to saline-injected mice (Fig 3A and 3C). To further characterize the role of TLR4 in RTEC response to LPS, we isolated primary cortical RTEC and grew them in defined media +/- 1 microgram /mL of LPS for 24 hours. As in the in vivo studies, LPS did not induce autophagy in RTEC from C57Bl/10ScN mice (Fig 3B and 3D). Therefore, LPS-induced autophagy in RTEC is dependent upon TLR4 signaling.

### Proximal Tubule Autophagy Limits LPS-Induced Kidney Injury

**Fig 4:**
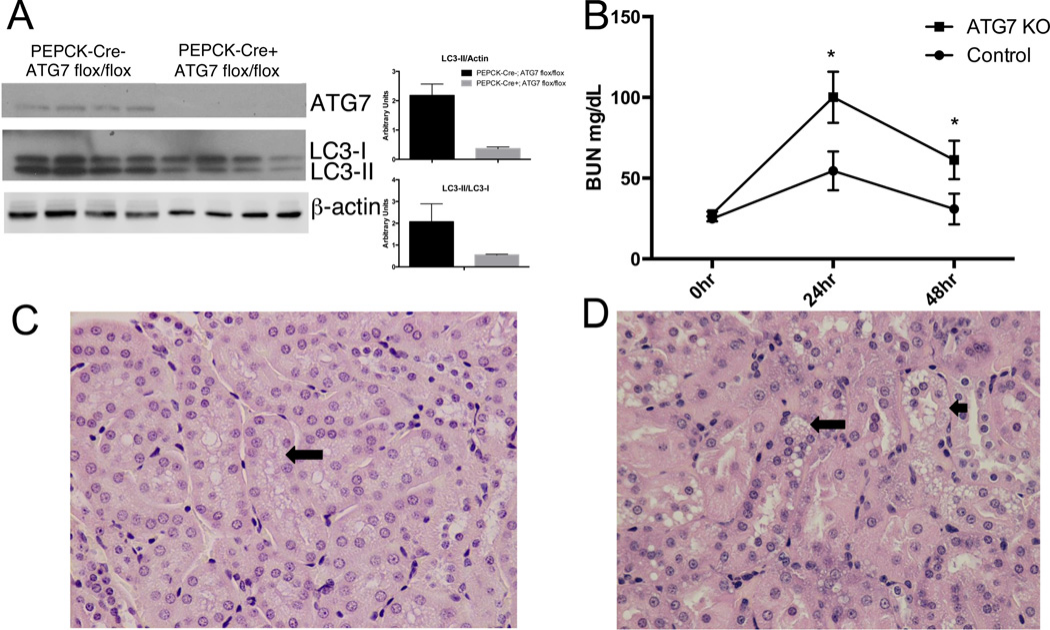
Proximal tubule-specific knockout of ATG7 increases severity of LPS-induced AKI: (A) Decreased ATG7 and LC3-II in cortical lysates from ATGKO *PEPCK-Cre;Atg7*^*fl/fl*^ mice compared to control *Atg7*^*fl/fl*^ mice. (B) Proximal tubule-specific deletion of ATG7 worsened LPS-induced AKI as reflected by higher BUN levels after 24 and 48 hours (* = p < 0.05 compared to controls at same time point). Administration of LPS induced only modest histological changes in control mice (C) with preserved architecture and only focal vacuolization (arrow), whereas ATG7KO kidneys (D) showed more diffuse indications of proximal tubular injury including substantial coarse vacuolization of proximal tubular epithelium (large arrow) with degenerating nuclei (small arrow).

To determine the functional consequences of impaired RTEC autophagy, we utilized a conditional knockout model. Mice with floxed ATG7 alleles [23] were crossed with mice expressing Cre recombinase under the control of the phosphoenolpyruvate carboxykinase promoter (PEPCK), which in the kidney is specifically active in proximal tubules [24]. The renal proximal tubules of the resulting *PEPCK-Cre;Atg7*^*fl/fl*^ mice do not express ATG7 [10], a critical autophagy gene participating in two different ubiquitin ligase-like processes in the autophagy pathway. As expected, ATG7 and LC3-II were decreased in RTEC from mice expressing both transgenes, consistent with the impairment of the basal renal tubular autophagy (Fig 4A). Intraperitoneal injection of LPS mimics conditions of fulminant gram-negative sepsis and induces both systemic and intrarenal immune responses resulting in AKI [25]. In this model, LPS rapidly enters the proximal tubular lumen after glomerular filtration [3]. To test our hypothesis that impairment of autophagy in the proximal tubule would worsen LPS-induced injury, *PEPCK-Cre;Atg7*^*fl/fl*^ (ATG7KO) and control *Atg7*^*fl/fl*^ littermates were injected *i*.*p*. with 15mg/kg of LPS. AKI was detected by measurement of blood urea nitrogen (BUN) in both groups, but was significantly higher in ATG7KO mice than in controls at 24hr and 48hr (100.1 and 61.3 vs 54.5 and 28.8, Fig 4B). While present in both groups, histological tubular injury was significantly more severe in ATG7KO than in control mice (p <0.05; Fig 4C and 4D).

### Altered Cytokine Expression in Autophagy Deficient RTEC

**Fig 5:**
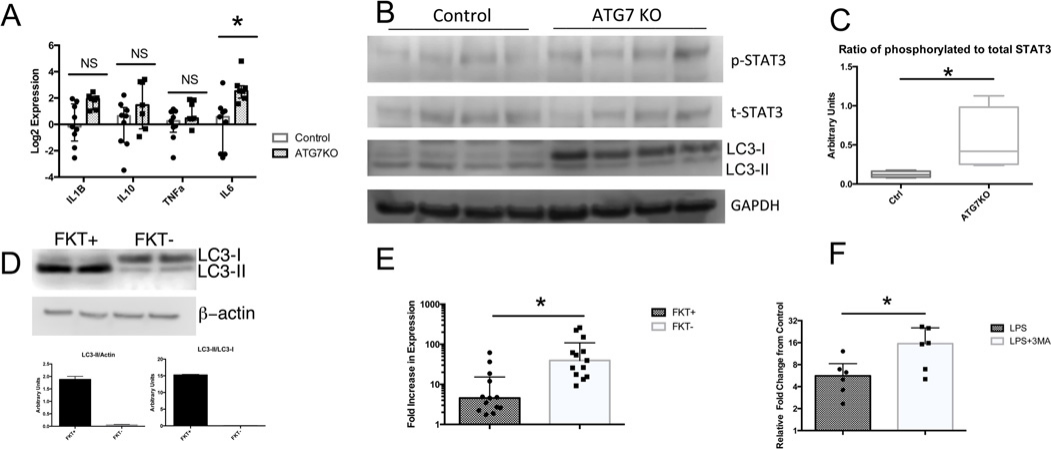
LPS exposure increases IL6 expression and STAT3 activation in ATG7KO RTEC cells. qPCR analysis demonstrated increased LPS-induced IL-6 expression in cortex of ATG7KO kidneys compared to LPS injected controls (A) which was accompanied by (B) decreased LC3-II and increased STAT3 phosphorylation. (C) Densitometric analysis of the expression of phospho-STAT3 relative to total STAT3. (D)Western blot of FKT- and FKT+ cells indicated that the ablation of Atg7 impaired accumulation of the LC3-II isoform in response to LPS (D). At the same time, qPCR analyses showed that the LPS induction of IL-6 was enhanced in the ATG7 deficient FKT- cell line (E) and in RTEC isolated from wild type mice co-incubated in the absence or presence of 5mM 3MA (F).

The etiology of sepsis-induced AKI is multifactorial, but dependent, in part, upon intrarenal TLR4 mediated cytokine induction [26]. To determine whether PTEC autophagy alters LPS-induced renal cytokine expression, we isolated RNA from LPS-injected control and ATG7KO kidneys and performed quantitative real time (qRT)-PCR for several cytokines known to have important roles in septic AKI. Interestingly, only IL6 was expressed at a significantly higher level in LPS-injected ATG7KO kidneys as compared to LPS-injected controls (Fig 5A). Because IL6-induced signal transduction occurs via Signal Transducer and Activator of Transcription 3 (STAT3), we evaluated the levels of phosphorylated STAT3 in control and ATG7KO kidneys. Western blot analysis of renal cortical lysates confirmed both impaired autophagy and increased STAT3 phosphorylation in ATG7KO kidneys compared to controls after LPS injection (Fig 5B and 5C).

To determine whether the increase in IL6 expression was related directly to tubular cytokine expression, we established a cell line from isolated tubules of *Atg7*^*fl/fl*^ mice (FTK+) and used them to create ATG7 deleted cells (FKT-) upon Cre expression (see Methods). As expected, FKT- cells had diminished functional autophagy compared to FKT+ as evidenced by the inability to form LC3-II (Fig 5D). After stimulation with LPS, FKT- cells demonstrated significantly higher induction of IL6 (Fig 5E), consistent with our *in vivo* results. To determine if pharmacologic suppression of autophagy would have similar effects, we isolated and cultured RTEC from wild type mice and treated them with LPS in the presence or absence of 5mM 3-methyladenine (3MA), an autophagy inhibitor. In agreement with what we observed in our cell lines, the inhibition of autophagy further increased the induction of IL6 in response to LPS (Fig 5F).

## Discussion

Our studies are the first to demonstrate that LPS induces TLR4-dependent autophagy in human RTEC *in vitro* and murine RTEC *in vivo*. Previous studies demonstrated that RTEC autophagy is cytoprotective in IRI and cisplatin induced AKI models [8, 11] and that the pharmacologic augmentation or inhibition of autophagy in septic AKI models mitigated or worsened kidney injury, respectively [13] [27]. Our data confirm the renoprotective effects of autophagy in AKI and extend the importance of this pathway to a sepsis model. While results from previous reports were based on pharmacologic modulators of autophagy that may interfere with other intracellular pathways, thus preventing a definitive conclusion on the impairment of autophagy, our study relied on the specific targeting of autophagy specific proteins.

One recent publication suggested that use of the autophagy inhibitor 3MA protected mice from LPS induced AKI by inhibiting autophagy. The conflict between this finding and ours highlights the importance of conditional knockout models. Chloroquine, for example, is a lysosomatropic drug that, amongst other effects, inhibits the terminal steps of autophagy and protects against septic AKI. However, the mechanism behind this protection is related to altered function of endocytic dependent innate immune receptors, rather than the effects on lysosomal function [28]. 3MA also has notable autophagy independent effects. Studies in peripheral blood mononuclear cells demonstrate that 3MA inhibits TNFα expression in response to LPS [29]. Accordingly, we observed a non-significant trend toward lower TNFα expression in 3MA treated RTEC incubated with LPS versus LPS alone (S1 Fig). However, this phenomenon was not reproduced *in vivo* or *in vitro* using genetic knockout models. Because TNFα is a major mediator of septic AKI, the decreased severity of AKI in 3MA treated mice may be related to an indirect effect such as its impairment of TNFα expression and independent of its inhibitory effects on autophagy induction [30].

A surprising finding in our studies was the specific difference in IL-6 induction as opposed to other prototypical inflammation associated cytokines. IL-6 is a known mediator of nephrotoxic injury and its absence prevents HgCl_2_ induced kidney injury [31]. While it is possible that the increased severity of LPS-induced AKI in mice with tubular specific knockout of ATG7 was due to increased IL-6 expression, our data only demonstrate an association. Further experiments are required to determine whether IL-6 actually mediates effects of autophagy in modulating the severity of septic AKI. Nevertheless, our work suggests a role for autophagy in regulating innate immune responses of the kidney that are likely to have importance beyond this LPS model of septic AKI. Our finding that autophagy had specific effects upon IL-6 expression that were independent from effects on other TLR4-regulated proinflammatory genes suggests that autophagy can affect transcription of specific genes, perhaps through degrading or preventing the degradation of specific transcription factors. In a larger context, innate immune signaling has been tied to the pathogenesis of multiple renal diseases and TLR4 signaling, specifically, has been identified as a mediator of diabetic renal disease [32]. As such, future work delineating the mechanisms through which autophagy regulates TLR signaling would have implications on both acute and chronic kidney disease.

Sepsis-induced AKI is a major cause of morbidity and mortality in hospitalized patients and there is a great need for research defining the intracellular pathways that are dysregulated in RTEC in the setting of sepsis. We provide strong evidence that LPS, an immunogenic bacterial cell wall component released during gram-negative sepsis, evokes protective autophagy in RTEC that is associated with altered expression of cytokines. Our work provides further evidence that autophagy is a viable therapeutic target for the prevention, mitigation, or treatment of acute kidney injury.

## Supporting Information

S1 Fig (TIF)
