# Supplementary figures and images for: Autophagy Limits Endotoxemic Acute Kidney Injury and Alters Renal Tubular Epithelial Cell Cytokine Expression
Source: PLoS One. 2016 Mar 18;11(3):e0150001. doi: 10.1371/journal.pone.0150001 (PMC4798771; doi:10.1371/journal.pone.0150001)

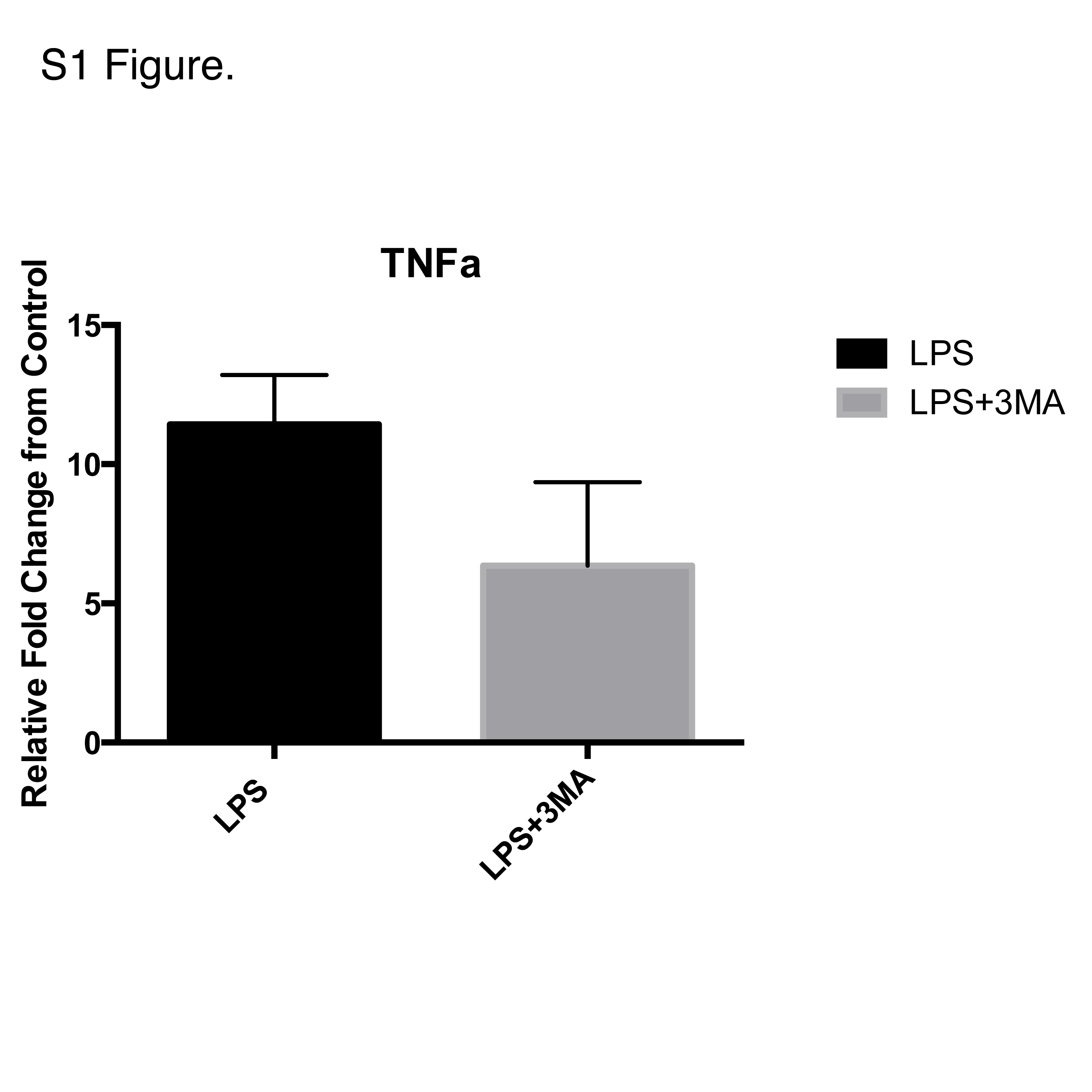

Supplement: S1 Fig — (TIF) [file pone.0150001.s001.tif]
